# Testing Times: The Place of the Citizenship Test in the UK Immigration Regime and New Citizens’ Responses to it

**DOI:** 10.1177/0038038515622908

**Published:** 2016-07-11

**Authors:** Bridget Byrne

**Affiliations:** University of Manchester, UK

**Keywords:** citizenship, citizenship testing, immigration, national identity, UK

## Abstract

Citizenship tests are designed to ensure that new citizens have the knowledge required for successful ‘integration’. This article explores what those who have taken the test thought about its content. It argues that new citizens had high levels of awareness of debates about immigration and anti-immigration sentiment. Considering new citizens’ views of the test, the article shows how many of them are aware of the role of the test in reassuring existing citizens of their fitness to be citizens. However, some new citizens contest this positioning in ‘acts of citizenship’ where they assert claims to citizenship which are not necessarily those constructed by the state and implied in the tests. The article will argue that the tests and the nature of the knowledge required to pass them serve to retain new citizens in a position of less-than-equal citizenship which is at risk of being discursively (if less often legally) revoked.

## Introduction

Regulation, practices and policy concerning immigration and citizenship have been in a state of continual adjustment and re-creation over the last 20 years in the UK, as well as other countries in Europe. This has included the introduction, in several countries,^1^ of regimes of testing applicants for citizenship or permanent residence. This article will argue that not only do the tests (and media responses to them) tell us about state constructions of citizenship, the nation and immigration, but that the accounts of those required to take the test can shed light on alternative claims to citizenship, belonging and inclusion. The European tests mimic the more established practices of testing for citizenship of the United States and Canada; however, they are much more recent introductions. The tests were introduced as part of what might be called a ‘citizenship turn’, where citizenship in general and naturalisation in particular have come under renewed focus in public debates and have resulted in many changes in what Engin Isin calls the ‘nationality-citizenship-apparatus’ (Isin, 2012: 567). This ‘turn’ in Europe over the last 10 to 20 years has occurred in the context of a questioning of multiculturalism and a return to more integrationist policies, with a particular focus on immigration and citizenship. Part of this shifting approach is a trend towards higher barriers for naturalisation, both in terms of the required number of years of legal settlement before immigrants are eligible to apply to naturalise and in the requirement to prove (often through tests) that integration has already been achieved (Goodman, 2010; Vink and de Groot, 2010). The European tests are distinct from the longer-established tests in the US and Canada because they have been developed within this specific climate of challenging multiculturalism and hostility to some forms of immigration. As Christian Joppke argues: ‘the coercive and punitive tone in some of Europe’s new citizenship tests and loyalty requirements is still noteworthy and, to repeat, a distinctly European innovation’ (Joppke, 2013: 3).

The introduction of the UK citizenship test in 2005 is a good example of this general European trend of the questioning of multiculturalism and its links to citizenship regimes. In the UK, political debates around immigration have been particularly intense over the last 10 years. These have been interwoven with debates which suggest ‘crises’ in multiculturalism and citizenship. British politicians, such as the Prime Minister David Cameron (along with other European leaders such as Angela Merkel and Nicolas Sarkozy), declared the ‘failure’ of multiculturalism (Lentin and Titley, 2011). This ‘European-wide moral panic about “difference”’ (Grillo, 2007: 980) is centred on the concern that there might be ‘too much’ difference for national unity and leads to a re-assertion of the need to preserve a narrow, majority-defined, national culture. In the period after 9/11, there has been a particular focus on religious difference and the construction of the Muslim ‘other’. Multicultural tolerance, and celebration of difference, has been blamed for fostering too much separation and of being weak in the face of cultural forces hostile to Christianity and western culture (Kundnani, 2014). In response to these fears, there has been a renewed focus on ‘British values’ – for example, in the recently introduced duty of schools to ‘actively promote fundamental British values’ through the curriculum.^2^ In addition to concerns about Islam and teaching, there has also been particular anxiety about the loyalties of those with dual citizenship. These concerns have a long history – including the infamous ‘cricket test’ suggested by Norman Tebbit in 1990 whereby supporting the English cricket team was posited as a test of integration.^3^ Since 2006, the Home Secretary has the power to remove British citizenship from dual nationals if it is perceived to be in the public interest, and there are moves to extend this power over those with no other citizenship, thus rendering them stateless.^4^ It is reported that between January and November of 2013, 20 people lost their citizenship.^5^

There has been some literature considering the creation and introduction of citizenship tests in the UK looking at the introduction of the tests, significant changes to it and the UK tests in international (particularly EU) comparison (Brooks, 2013; Cooke, 2009; Etizioni, 2007; Kiwan, 2008a; Osler, 2009; Wright, 2008). However, the experiences and views of those required to take the test has been notably absent in this scholarship. In addition, much of the literature examines the details of the differences between EU tests without exploring the implications of testing for our understanding of citizenship regimes or practices. They often fail to examine the position of tests in wider representations of citizenship, national identity and naturalisation. This article, based on interviews with new citizens, will be the first to explore what those who have been obliged to take the test think about both their content and the requirement to take it. It will also situate the tests in public responses to them. The first section will detail the research which this article contributes to and the next will provide an account of the place of the citizenship test in legislation and media discourses of citizenship, immigration and nation. It will argue that the tests can be seen as an attempt to reassure (Fortier, 2008: 101) public anxieties about immigration by requiring a display of the suitability and propriety of potential citizens who have shown a commitment to learning and the potential to integrate. Nonetheless, the media coverage shows that there remain continual anxieties that the test is not sufficiently hard or does not contain the right questions or knowledge. The article will argue that the tests, and the nature of the knowledge required to pass them, serve to retain new citizens in a position of less-than-equal citizenship which is at risk of being discursively (if less often legally) revoked. The article will then consider the views of new citizens who have taken the test. It will demonstrate that many are aware of their part as probationary citizens, who need to reassure ‘the British’ of their fitness to be citizens. At the same time, the article will explore how some find various means by which to contest this positioning, in part through asserting claims to citizenship which are not necessarily those constructed by the state and implied in the tests. In this way, drawing on both theoretical approaches to citizenship and the empirical experience of new citizens, the article suggests how citizenship testing is an important site of the remaking of citizenship with implications that go beyond the more limited frame of immigration regimes.

## New Citizen Perspectives on Taking the Test

This article comes out of a larger study (Byrne, 2014) which was focused on citizenship ceremonies in both the UK and abroad. The study involved analysis of the texts of the local welcomes given at ceremonies in the UK (Byrne, 2012) as well as, in 2010, observation of ceremonies in 10 locations around the UK as well as in six other countries.^6^ At each observation in the UK, a few of the new citizens were also interviewed either on the day, or soon after the ceremony. The interviews took place in the interviewees’ homes, in spaces provided by the registrars, or in local cafes, and covered questions about new citizens’ experiences of coming to Britain and of applying for visas and citizenship, which included questions about the Life in the UK (citizenship) Test. Whilst researcher access to the ceremonies was generally straightforward, new citizens were often reluctant to be interviewed. This reluctance may stem from the fact that the route to citizenship is long and difficult for many and frequently involves mixed feelings. In addition, despite the reassurances given, there may have been fears that I was somehow connected to officialdom, as represented by the registrars and the Home Office.

Thus, those who were willing and able to be interviewed for this research (30 in total) represent a particular subset of the new citizens, who share some characteristics. Alongside a willingness to be interviewed, they also were (with a couple of exceptions for whom translation was provided) those who were sufficiently confident in their English language skills. The interviewees came from 19 different countries of origin and, apart from the absence of interviewees originally from the Philippines, broadly match the range of countries of origin of those applying for citizenship in the UK.^7^ The age of the interviewees ranged from young adults to late 60s. Twice as many women than men agreed to be interviewed. There are slightly more women naturalising each year in the UK than men, but not enough to account for this difference.^8^ Some women were not in paid employment, and thus more available for interviewing. There was also likely to be a gendered response in that women were more likely to feel sympathetic to me as another woman and perhaps also more comfortable with the prospect of a one-to-one interview.

At one level, the interviewees can be seen as an elite group of migrants – those who are able to come to Britain legally (or establish their legality) and stay long enough to qualify for citizenship. However, it is also important to note the many diverse routes into citizenship that they have taken. Their diversity is in part shown by their modes of entry to Britain, as well as differences in length of residency before they had applied for citizenship. Many of those who were interviewed had applied for citizenship as soon as they were eligible (usually five years, although shorter for those on a spousal visa). However, for others there had been considerable delay before they applied. The decision to apply long after they had been eligible reflected shifts in their own orientation towards British citizenship. It was also a product of changes in the citizenship regimes which made it seem more urgent to take up citizenship before future alterations made it more expensive to apply for citizenship, or even made them ineligible (see Byrne (2012) for further discussion). In terms of entry routes into Britain, there were wide differences in experience. For some of the interviewees, the process was mediated by the multinational corporations which had brought them to Britain for work and which also managed the visa application process. Other interviewees initially made use of ‘ancestral’ visas which give individuals from Commonwealth countries the right to stay in Britain for three years on the basis of having a grandparent born in Britain. These are largely available to white individuals (Tyler, 2010). There were several interviewees who had come on ‘spousal’ visas and, for some, this was a very isolating experience (see Byrne, 2012). Several of the interviewees had come into Britain as asylum seekers and therefore, unlike others, did not necessarily have a choice of where to settle. Asylum seekers cannot work and have an uncertain status in Britain until refugee status is granted – often a long and difficult process of establishing claims to a sceptical state (Pannett, 2011).

Perhaps inevitably, as a result of the self-selection that is involved in agreeing to be interviewed, almost all the respondents I spoke to had taken the test route rather than the ESOL (English for Speakers of Other Languages) route which was available at that time and which involved attending a citizenship-focused English language course, with no final exam. In other words, although their standard of English varied, it was good enough for them to take the test. However, some had failed the test at least once (not always because of issues around language). Before considering the participants’ views of the test, the next section will consider the context in which the tests were introduced. As will be discussed below, elements of the context were also understood by the participants who were aware of the political and media debates around citizenship and immigration as well as the role that the tests played in them.

## Testing Citizenship, Testing Nation

The introduction of the new citizenship regime in the UK, including the citizenship tests and ceremony, can be seen as products of ongoing political debates about the nature of Britishness and citizenship.^9^ Politicians from both the left and right of the political spectrum in Britain have engaged in debates around the meanings of nationhood, often in the context of an argument about a supposed citizenship ‘crisis’ (Turner, 2014). The concern frequently voiced is that the British do not know what citizenship and Britishness mean. This argument was particularly present, for instance, in response to civil disturbances in Oldham and Bradford in 2001 and in the declared ‘War on Terror’. In the context of the latter, some basic terms of the relationship between the citizen and the state have been redrawn (for instance, in the control orders in which the state restricts individuals’ mobility and subjects them to extra scrutiny without recourse to a trial). The New Labour Government proposed various solutions to this crisis. These included the introduction of citizenship studies in schools (2002); the bi-annual ‘citizenship survey’ begun in 2001; and attention given to the endowing of citizenship to new British subjects. With regard to citizenship tests, this can be seen as a process of ‘exclusive-inclusion’ (Turner, 2014), where the inclusion of some also represents the exclusion of others – what might be called the ‘anti-citizen’ (Barbero, 2012). The test established both a barrier to inclusion and also a normative ideal of what a citizen – and a new citizen in particular – should be (or at least what they should know).

The idea of citizenship testing was introduced in the Government White Paper *Secure Borders, Safe Haven: Integration with Diversity in Modern Britain* (Home Office, 2002). In the Paper, the emphasis was on the need for potential new citizens to have a good grasp of English, with less discussion of what was called ‘education for citizenship’. Nonetheless, David Blunkett (Home Secretary between 2001 and 2004), in his forward to the White Paper, proposed that ‘those coming into our country have duties that they need to understand and which facilitate their acceptance and integration’ (Home Office, 2002: 5). The first test, introduced in the UK in 2005, was based on the book *Life in the United Kingdom: A Journey to Citizenship*, which was written by Bernard Crick, an advisor to the Labour Government, who was also involved in the introduction of citizenship education in schools. Passing the test was initially only required for those applying for citizenship, but it is now required of applicants for permanent residency.

Dina Kiwan, who was a member of the ‘Life in the UK’ advisory group, has argued that the tests should not be seen as signalling a more ‘restrictive’ approach to citizenship and integration but should rather be set in the context of the newly developing citizenship education (Kiwan, 2008a: 61). At least in part, this meant, according to Kiwan, that the test was regarded as ‘an early step in the journey towards citizenship, rather than the test signifying a static “badge” of honour at the end of a journey’ (Kiwan, 2008a: 69). This accords with the statements at the time of the Home Office immigration minister Tony McNulty who explained that the official objective of the test was to ‘be helpful to integration’. He also argued: ‘This is not a test of someone’s ability to be British or a test of their Britishness. It is a test of their preparedness to become citizens (quoted in Whitehead, 2006: 8). However, as we shall see below, the test *is* often regarded in popular discourse as a test of Britishness.

Kiwan stresses that the Life in the UK advisory group had integration (which she fails to define) as their primary goal in proposing a new approach to the process of naturalisation. However, she also details how some of the earlier proposals which accompanied the test (such as for ‘participative opportunities for applicants’) were not taken up by the Government, which limits their integrative capacity (Kiwan, 2008a: 69).

Whatever the intention of the advisory group, the requirement to pass a test constitutes an impediment to settlement and citizenship. In 2013, the regulations were altered so that passing *both* a language and a knowledge test was a requirement for applicants for permanent residency as well as for naturalisation. The third edition of *Life in the United Kingdom*, published in 2013 by the Coalition (Conservative and Liberal Democrat) Government, placed more emphasis on British history and achievements and reflected an increased focus on integration and participation, thus reflecting the shift to ideas of ‘earned’ citizenship, where rights can only be granted once they have been demonstrably earned. As Mark Harper MP, Minister for Immigration, explained: ‘The new test rightly focuses on values and principles at the heart of being British. Instead of telling people how to claim benefits, it encourages participation in British life’ (quoted in Brooks, 2013: 23). This shift in emphasis has also involved the removal of practical information, such as how to get medical assistance through the NHS, details of educational qualifications and reporting crime (Brooks, 2013).

The tests have attracted considerable media attention in the UK. Notably, much more than the more celebratory citizenship ceremonies which were introduced a year before the first test was published. Whilst the first citizenship ceremony in the London Borough of Brent (attended by Prince Charles and David Blunkett in 2004) did get national media coverage, after this there has been very little discussion of the ceremonies in the media. As a result, public awareness of what are officially private ceremonies is low. In contrast, the introduction of the test promoted widespread coverage (including many chances for readers or viewers to see if they could pass the actual tests or spoof versions). This attention has been repeated whenever the test has been changed or updated. Despite being called officially the *Life in the UK Test*, it is frequently dubbed by the media as the Britishness test.^10^ On its publication, the test was criticised variously for being too hard, too easy and for failing to test candidates on history. The original book *Life in the UK*, on which the test is based, has a section titled ‘The making of the United Kingdom’, which is included for interest, but which is not part of the test. James Slack of the *Daily Mail* deplored the situation where: ‘it is possible to become a citizen while having no knowledge of Churchill, the two World Wars, Nelson or the Battle of Waterloo’ (Slack, 2005). The question of the inclusion or exclusion of history has continued to dog the test. This reflects similar controversies about the teaching of history in schools and reflects the importance of history in national narrations (Bhabha, 1990; Byrne, 2012; Phillips, 1998).

The dominant voices in public discussions of the test were those expressing concerns that the test was too easy. Here it can be seen as failing to reassure the neurotic citizen (see discussion below) that sufficient barriers have been placed around the borders of citizenship. For example, Tom Whitehead, writing in the *Daily Express*, argued that: ‘People who can hardly speak English could be given citizenship without even sitting the exam’ (although it is only after several paragraphs that he explains that they would have to undertake an ESOL course instead). Whilst on the one hand reporting that ‘Only one in 20 test tutors who sat sample exams passed them’, it also complained that ‘it only grills migrants on day-to-day life and asks them nothing about the nation’s proud history’ (Whitehead, 2006: 8). The guide for the test, and therefore the test itself, has been repeatedly rewritten since it was introduced. This can be seen as part of an ongoing effort to ensure that the test represents the right level of obstacle to be overcome, or provide sufficient education, and perhaps also to respond to media critique. The next section will explore the new citizens’ views of the test, which, in part at least, are also shaped by awareness of this broader media debate. In particular, many interviewees understood the test as contributing to the reassurance of the broader citizenry that they had made a serious commitment to becoming citizens of the UK and were fit to do so.

## New Citizens’ Perspectives on the Tests

### Gaining Knowledge

For some of the respondents, the citizenship test did work in the way intended by the advisory group. Revising for the test gave them practical information or an awareness of history that they found useful and interesting. As Makena, a nurse from Kenya, explained:

I got a lot of information that I didn’t know. I was told a lot of things: how to do things that I didn’t know how to do; where to get information; how the government works; how the community is – this kind of thing.

The usefulness is a second order of utility – not essential to everyday life in the UK, of which the respondents already had extensive experience. That the knowledge gained in the test was not critical and was thus ‘forgettable’, was underlined for many by the respondents’ awareness that many Britons-by-birth could not have passed the test. Helen, a white woman from South Africa, said she found the test ‘daunting’ and added: ‘Actually, you know, you end up having a good time. I think and I know a bit more than the British people know about their own country.’ Bernard told how he had rung up to arrange to sit the test and was encouraged to study for it. He explained to the woman at the test centre that, as an academic who had lived and worked in Britain for 30 years, he did not think he would have trouble passing the test. But she again insisted that he should study. He duly bought the guide book and found that she was right – he would not have been able to pass without that extra study. For Melody, who had strong attachments to her adoptive city, there was pleasure in knowing more than local ‘Scousers’ born in Liverpool knew. However, this sense of extra knowledge that they need to gain, which just living in the country would not bring, underlines the terms of entry to citizenship. It is not enough for migrants to be *just like* citizens-by-birth – they must demonstrate *extra* commitment and capacity. Elizabeth Badenhoop (2014) refers to this ideal-type as the ‘super citizen’ which new citizens are required to perform at various parts of the process of becoming citizens.

Hamed, who had come to Britain from Iran as a child, was aware that an extra commitment was required from potential citizens and he felt that this was fair:

To be honest, I think that it’s very acceptable to ask people who want to take British Citizenship to do the test. It’s only your way of saying that I’ve done a bit [of] research to show that I am actually interested in this country’s laws and background, so it’s very acceptable and even if it was a little bit more advanced it would still be acceptable.

In this way, Hamed reflects the idea of liberal citizenship, where the would-be-citizen demonstrates his or her commitment and worth through the time and effort taken in learning for the test (Turner, 2014). Hamed clearly expresses the government’s rationale behind the test; however, his positive view of the test was a minority position among the interviewees.

For some respondents, such as Amna from Bangladesh, the fact that passing the citizenship test requires knowledge of facts that those who have citizenship by birthright do not know, **can** lead to a sense of injustice:

In the beginning I felt ‘Oh, what’s the point of having to learn all of those things because those people who were born in Britain, were brought up in Britain, they don’t know anything about those [things]’.

However, learning about the history of immigration in Britain from the *Life in the UK* booklet gave her a different sense of her place in Britain:

After that I realised that this helps me feel more like – when I understand that when – back to the fifties when people are invited in England after Second World War, all those things and they helped Britain to build in different ways. … then I will feel, I never feel that I am someone from a different country or somewhere outside. … Knowing all those informations from that book, I feel that I’m not here without any reason because they have invited us from previous [times].

Learning this history gave Amna a longer historical view from which to understand her immigration to Britain and to contest how she, as an immigrant, is represented.

### Citizenship Tests and the Neurotic Citizen

As well as their interest in the historical narratives produced in the test booklets, many interviewees had a high level of awareness of and interest in political debates around immigration. Claire explained: ‘It was quite funny. We’re not interested in Australian politics [but] we really follow it here.’ Migration itself can bring with it increased participation and interest in citizenship practices. Moving from one country to another, and having to negotiate rules and regulations that control mobility, may make individuals more aware of the importance of politics and policy. The new citizens were aware that the tests had been introduced in a climate of opposition to immigration. Ghedi suggested that there was an element of manufacturing of anxiety around immigration:

It’s the politicians using it as a kind of, you know, something to show the poor man on the floor, you know, saying, ‘yeah, they’re coming in, and we’re going to do something about it’ instead of giving any jobs … it’s been becoming kind of a bit stronger and stronger and stronger lately but … it’s in the society; the people are always afraid of immigrants coming in. … the hostility, it’s always there.

Ghedi’s account reflects on the production of a ‘neurotic citizenship’ in which, according to Engin Isin, the neurotic citizen ‘governs itself through responses to anxieties and uncertainties’ (Isin, 2004: 223). Thus, the tests are part of a process of reassuring citizens that access to citizenship is controlled and the country is ‘safer’.

Similarly, Claire spoke about her work colleagues and their hostile attitudes to immigration, particularly in the context of the run-up to the 2010 election. Claire felt sure that her colleagues did not regard her (a white middle-class woman from Australia) as a migrant. She described what she assumed was their imagined figure of a migrant: ‘Muslims that migrate to a country and then have 12 children on benefit. You know – that kind of thing.’ Some interviewees suggested that they were perhaps not *those* types of immigrant who they imagined the test (as either disciplining or pedagogical technique) to be aimed at. This assertion might stem from the length of time they had lived in the country or evidence they had other claims of affiliation to Britain (both will be discussed below).

Running through many new citizens’ responses to the test (in common with those of the media) is a tension about what the tests are intended to achieve and whether they meet those aims. If they are primarily intended as a barrier to be overcome as a form of restriction on applications, then perhaps the knowledge gained is irrelevant. It is just ‘book knowledge’ as one interviewee called it: ‘it’s just for the test and then after it’s gone’. However, some valued knowledge which equipped them as citizens. Furthermore, Simone suggested that it might also foster a more confident sense of belonging: ‘At least you will be knowledgeable. If someone is asking you about the country and you are just like a dumb foreigner. [After learning for the test] You know what you are talking about.’ This transition from ‘dumb foreigner’ to worthy citizen is clearly important for Simone. Nonetheless, passing the test provides little guarantee that these claims will be recognised more widely.

### Citizenship Acts?

The majority of respondents had reservations about the test, which focused less on the requirement to take it and more on its content. What is interesting about the critiques is the way in which respondents use this moment of discussing the ‘testing’ of their readiness to be citizens or long-term residents as an opportunity to assert other forms of belonging or affiliation to Britain – in what could be understood as citizen acts. For Isin and Nielson (2008), ‘citizenship acts’ are those actions and practices which trouble the borders of citizenship through subjects *acting as* citizens even where the state may not recognise them as such. This concept could be extended to consider the citizenship *claims* made by those whose citizenship is often contested or seen as provisional. The existence of the tests positions those who take it as outside of citizenship, until they have proved their worth by passing. However, in their critiques of the tests, new citizens often emphasised their *already existing* knowledge and claims to citizenship. For many, what was inappropriate about the tests was that the information given appeared to assume a very low base of knowledge, ignoring their long residence in Britain. All the new citizens had lived in the country for at least five years before taking the test and many had been in the country for much longer. Ghedi, who had come to Manchester from Somalia as a child, had taken many years to get round to applying for citizenship, despite being eligible for it. He laughed when I asked if the information he’d had to learn to pass the test was useful:

Yes and no. Yes, if you have not lived in this country or you lived in a cave in this country for six years [laughs] it would be useful, but most of the people, after having lived in this country for six years, would know all those things anyway. There are certain things that you have to memorise, like dates and percentages of minorities, ethnic minorities. … those little details which I completely forgot by the way.

Ghedi suggests that the test assumes a high degree of ignorance by testing knowledge of information which would inevitably be absorbed by living in the country (rather than in a cave). This critique of the test was common among the respondents. Thus they contest the idea that the process of learning for the test improves their ability to engage in British society.

Rada, from Bulgaria, put it even more strongly:

It was an extremely stupid test. Extremely. I have studied, I have [taken] exams. … I always admired the English education system. That was extremely stupid because I would rather have something to know about, something about English history, something that we can just be proud of. All the study was: ‘Who came from which country, how many people?’ ‘What percentage of them are Muslim or Christian?’ I’m sure that changed every day.

Rada emphasises she does not find the test difficult. She is a responsible and disciplined liberal subject. She also states her admiration for (and implicitly her knowledge of) the English education system and her desire to be provided with knowledge that would make her ‘proud’ of her Britishness. Her objection is to the information that had to be memorised, which is of little practical use and which will rapidly become out of date. Rada is not alone in highlighting the need to memorise the percentages of people in the UK following different faiths as a particularly useless thing to learn. As Neela from India said:

Yeah, but then they couldn’t make sense like, they’re a lot of figures about what population and what percentage and it doesn’t make any sense to mark those you know, to get those figures in your head because it’s just for that test and then after it’s gone.

This is not knowledge that is really required of, or useful to, a citizen. In addition, in some interviews, there was a resistance to the idea that Britain’s multicultural richness has been reduced to a statistical pie chart and suggestion that the test could involve more useful or meaningful learning. In contrast to statistics, the historical elements of the test booklet were often of most interest to respondents. As Rada asserted: ‘people … should know their history’. But she objected to the way immigrant communities were depicted through the accounts of the low-skill jobs they came to work in: ‘it was a long time ago, it doesn’t matter anymore’.

In what might be regarded as further citizenship acts, the respondents, whilst at times suggesting that *others* might not have sufficient knowledge to be considered for citizenship, asserted that *they* had connections and knowledge which made the test inappropriate or unnecessary. For instance, Claire suggested that, as a citizen of the Commonwealth (and possibly implicitly as a white person of Christian heritage), she already, almost instinctively knew much of the relevant cultural information (if not the exact percentages of religious groups):

I don’t think it’s designed for people coming from a Commonwealth country background. I mean it was good, I wouldn’t have passed it if I hadn’t studied for it, so I think some of the stuff was of benefit to me and some of the stuff was just a given. You know, what’s the day after Christmas? (A) Valentine’s Day; (B) Mother’s Day; you know, so a mixture of those really simple questions and then some which I didn’t know: ‘what percentage of the population is Catholic?’ Do I need to know? I don’t know that I really do, but I had to study for it. … But I could see why it would be of benefit to someone coming from a culture that is not similar to what is in the UK. It is of benefit to understand what the distribution of the population is, what the primary source of immigration over the period is. To understand what the make-up of the country is; and then there was some practical stuff in there about benefits, child labour, which I would imagine would be quite different in other countries as well.

Similarly, Prakash asserted his shared culture and history with Britain: ‘You see, Indians are nearly a 100 years in Britain’. This shared culture, and his involvement in local community cultural events meant that ‘as far as I am concerned, I am already a citizen’ even before the state’s official endowing of citizenship. This also shaped his responses to the racism that he and his wife had experienced: ‘We can’t get everything right, no? We can’t teach everybody the right things, no?’ Prakash asserts his sense of belonging through the use of the pronoun ‘we’. Later, in discussing the particular situation of Northern Ireland where he lived, he returned to this idea of common responsibility in a way which could be regarded as a citizen act:

Most of the people in Northern Ireland came from different parts of England … People should know the past. We have to teach the younger generation the past … it’s all migration … people are moving around.

Prakash again stakes his claim to be regarded as part of the ‘we’ of the citizenry, even before having officially become a citizen. But his restatement of both the importance of understanding history and the ignorance of many of those who have unquestioned claims to citizenship serves to highlight what is at stake in a test which is only set for those who come to Britain as migrants. Underlying his critique is a suggestion that the test is aimed at the wrong people and that it may be the citizens-by-birth in Britain who need to be educated on the histories and realities of migration.

## Conclusion

Testing citizenship can tell us about understandings of nation and citizenship more generally. The tests are part of the continual reconstruction and redefinitions of immigration and citizenship which have characterised British (and other European) policies over the last 50 years. In the UK, the tests characterise a particular moment in a narration of the nation-state with a re-assertion of ‘British values’ and an attempt to invigorate a more assertive citizenship. This development has been informed by political hostility to immigration and against the backdrop of the ‘War on Terror’ which has raised anxieties about the loyalties of both immigrants and their descendants. Scholarly focus on debates around integration, multiculturalism and citizenship tends to focus on the state and its modes of exclusion and inclusion. Very little attention has been given to the accounts of those who successfully navigate the state’s nationality-citizenship-apparatus (Isin, 2012). Yet these accounts can tell us both about the experience of undergoing this transition as well as the possible opportunities for contesting anti-immigration narratives and the construction of new citizens as outsiders to the society they have lived in for several years.

The introduction of national knowledge-based testing in the UK in 2005 followed a trend which was shared by other European countries. The test was embedded in a whole range of government policies towards immigration, citizenship and multiculturalism which were characterised by a return towards the language of integration. Anxieties around immigration have often been framed in terms of a lack of commitment to ‘British values’ or ‘British culture’. This formulation tends to rely on a notion of British culture as homogeneous and knowable or fixed. One thing that the media responses to the various tests have shown is that there is no commonly agreed version of what is required for someone to be considered ‘ready’ for British citizenship. Nonetheless, despite the controversies, the existence of the tests for applicants for permanent residence and citizenship may work to shore up some public anxieties both around migrants’ suitability for and commitment to integration. The very existence of testing may both construct and reassure the ‘neurotic citizen’ that the boundaries around the nation and who is to be considered a member of the nation are sufficiently high and arduous. Or, at times, they may have the opposite effect by appearing to be too lax or meaningless.

If tests are designed to facilitate integration, then it is a one-way model of integration imagined where all the work of adaptation is done by the incomers. Other citizens do not get tested on their readiness for integration, despite what Prakash called their ‘sad lack of knowledge’.^11^ This article has shown that, in the UK, tests were introduced (and have been modified) at times when immigration is highly politicised. In addition, the restrictive impact that the tests have on permanent settlement and citizenship is demonstrated by the numbers who fail the citizenship test – from 2010 to 2013, the failure rate for the Life in the UK test averages at 22 per cent.^12^ These effects are well understood by those who are required to take the test. There is a consistent pattern of an increase in applications for citizenship both before the introduction of the test in 2005 and later ahead of changes to the test (e.g. applications for citizenship rose by 17 per cent just before the changes to the test introduced in October 2013).^13^ Indeed, Bernard Crick himself explained that he had written *Life in the UK* in haste in order to ‘forestall a rush by settled immigrants to apply under the old, ludicrously inadequate regulations’.^14^ Whilst few of the interviewees objected strongly to the requirement to take a test, they did take the right, as citizens, to contest both the content and utility of what they had been required to learn. Thus, this article has argued that those who are obliged to take the test are well aware that they are required to acquire and display knowledge that most other citizens do not know. This leads some to contest the terms on which the tests are based.

New citizens come from a range of backgrounds and the citizenship tests can seem too easy or too hard. For many of those interviewed in this research, there was an awareness of how the tests might both produce and reassure the neurotic citizen. Thus, the tests represent a hurdle to be overcome, a demonstration of commitment and effort and a performance of the right kind of citizenship (or even ‘super citizenship’). New citizens were also aware of the ways in which immigration was frequently portrayed in Britain as a threat which had to be controlled. Whilst there was not extensive criticism of the need to take the test, there was nonetheless resistance to the need to learn facts – particularly demographic facts – that had no real use in everyday life and which the majority of citizens-of-birth do not know. Furthermore, asserting how the information in the citizenship test is inappropriate or meaningless can also be a route to establishing how the new citizens *already* had knowledge about British society and culture which had been built over long years of residence – and by a series of cultural and historical linkages (including those forged in Empire) which are not always acknowledged.^15^ The article has argued that listening to the targets of the policy (those taking the tests) provides a critique of testing from an original and particularly productive angle. In particular, new citizens’ responses to testing open up a series of assertions which can be understood as ‘citizenship acts’. These assert different principles of citizenship than those suggested by the state-defined tests. These claims are made on the basis of recognition of the ways in which extended periods living and working in the UK brings with it both knowledge of society and desires for belonging.

At the time these respondents took the test, the historical element of the guide book for the test did not form part of the test. Whilst the new citizens had little to say about the idea of British values or Britishness which the introduction of the test was intended to promote, history was of considerable interest to them. Some contested the way that history – particularly of immigration – was told. However, for others the account of different waves of immigration into Britain enabled them to also see their own migration in a different light, as part of a longer history of immigration into Britain. Thus, gaining knowledge of the UK histories of migration through preparing for the tests enabled some new citizens to shape other claims to citizenship outside of state narratives.

Citizenship tests – reserved only for those seeking permanent residence naturalisation – undermine the idea that they can become citizens ‘like any other’ once naturalised. The re-imagining (or retreat from) multicultural Britain, of which the tests are a part, ensures that new citizens remain at risk of being cast as ‘dumb’ (as one respondent put it) or even dangerous foreigners whatever their legal status. Nonetheless, the new citizens’ responses to the test explored here demonstrate citizenship claims based on other forms of knowledge and connection. These citizenship acts suggest an alternative model of citizenship, belonging and inclusion. The exploration of new citizens’ lived experience of migration and settlement, as well as their experiences of naturalisation, thus enables new ways of understanding claims to citizenship. It also provokes a rethinking of how the nation and belonging are constructed.

## Notes

1. In 2010, Sara Wallace Goodman (Goodman, 2010: 15) lists Austria, Denmark, Estonia, Germany, Hungary, Latvia, Lithuania, Moldova, the Netherlands, Romania and the UK as European countries which have introduced citizenship tests which include country knowledge (as opposed to language tests). Goodman cites France, Slovakia, Greece and Switzerland as using informal interviews to assess knowledge.

2. See https://www.gov.uk/government/news/guidance-on-promoting-british-values-in-schools-published (accessed 3 January 2015).

3. This example illustrates the tension and often confusion presented by national identity in Britain – a nation-state made up of four nations.

4. http://www.thebureauinvestigates.com/2014/01/30/home-secretary-pushes-through-controversial-powers-to-make-people-stateless/ (accessed 3 January 2015).

5. http://www.thebureauinvestigates.com/2013/02/26/medieval-exile-the-21-britons-stripped-of-their-citizenship/ (accessed 3 January 2015).

6. The USA, Canada, Australia, Ireland, the Netherlands and Italy.

7. In 2012, the largest groups by citizenship naturalising in the UK were: India (15%); Pakistan (5%); Nigeria (5%); and the Philippines, South Africa and China (4% each) (Blinder, 2013: 2).

8. In 2012, there were 41% adult women; 39% adult men; and 20% children naturalising in Britain (Blinder, 2013: 6).

9. This also includes increased focus on citizenship education. See Kiwan (2008b, 2008c, 2013).

10. As just one example, see *The Independent*. http://www.independent.co.uk/news/uk/home-news/more-than-half-of-young-people-fail-britishness-test-9684445.html (accessed 11 September 2014).

11. Although developments in citizenship education (despite not directly dealing with multicultural integration) do show a desire to overcome this (Kiwan, 2008c).

12. https://www.gov.uk/government/uploads/system/uploads/attachment_data/file/271513/Stats_for_FOI_29392_Life_Test_in_UK_for_questions_1_2_15-01-2014.pdf (accessed 15 July 2014).

13. https://www.gov.uk/government/publications/immigration-statistics-january-to-march-2014/immigration-statistics-january-to-march-2014 (accessed 15 July 2014).

14. http://www.theguardian.com/commentisfree/2006/may/11/post80 (accessed 15 July 2014).

15. As I have shown elsewhere (Byrne, 2012), in citizenship ceremonies there are often accounts of the history of Britain in the speeches. However, the history of Empire and Commonwealth are largely absent.
